# Supplementary material for: Effect of Flaxseed Supplementation on Milk and Plasma Fatty Acid Composition and Plasma Parameters of Holstein Dairy Cows
Source: Animals (Basel). 2022 Jul 26;12(15):1898. doi: 10.3390/ani12151898 (PMC9332015; doi:10.3390/ani12151898)
Supplement: Supplementary file 1 [file animals-12-01898-s001.zip › animals-1759812-supplementary.pdf]

Supplementary Table S1. Composition of experimental diets (% DM basis).

Items	Treatments[1]		
	CK	WF	GF
Diet ingredient, % of DM			
Corn Silage	21.51	21.86	21.86
Alfalfa hay	8.76	8.90	8.90
Alfalfa semi-dry silage	3.61	3.67	3.67
Wrapped straw	2.44	2.48	2.48
Corn	26.18	21.56	21.56
Corn flakes	2.77	2.82	2.82
Wool cotton seed	3.88	1.97	1.97
Orange peel granule	1.94	1.97	1.97
Soybean meal	12.42	11.8	11.8
Puffed soybeans	2.22	2.26	2.26
Cotton meal	3.12	3.17	3.17
DDGS	2.85	2.90	2.90
Bran	1.08	1.10	1.10
Fat powder	1.71	1.55	1.55
Flaxseed	0	6.38	6.38
Premix[2]	5.51	5.60	5.60
Total	100	100	100
Chemical, % of DM			
DM	51.18	51.27	53.58
CP	17.19	17.48	17.48
ADF	17.99	19.30	17.08
NDF	63.15	65.70	61.23
NE_L[3], Mcal/kg of DM	1.91	1.94	1.94

DDGS = distillers dried grains with solubles; DM = day matter; CP crude protein; ADF = acid detergent fiber; NDF = neutral detergent fiber; NE_L = Net energy for lactation.

[1] CK = dairy cows fed a basal diet (without flaxseed); WF = dairy cows with whole flaxseed diet (whole flaxseed 1,500 g per day); GF = dairy cows with ground flaxseed diet (ground flaxseed 1,500 g per day).

[2] Premix (per kg of DM): a minimum of 313,500 IU of vitamin A, 104,500 IU of vitamin D, 5,000 IU of vitamin E, 780 mg of Cu, 780 mg of Fe, 780 mg of Mn, 3,900 mg of Zn, 30 mg of Se, 50 mg of I, and 65 mg of Co.

[3] Calculated value (based on China Standard NY/T 34; People's Republic of China, 2004).
